# Paliperidone Inhibits Glioblastoma Growth in Mouse Brain Tumor Model and Reduces PD-L1 Expression

**DOI:** 10.3390/cancers13174357

**Published:** 2021-08-28

**Authors:** Yu-Shu Liu, Bor-Ren Huang, Ching-Ju Lin, Ching-Kai Shen, Sheng-Wei Lai, Chao-Wei Chen, Hui-Jung Lin, Chia-Huei Lin, Yun-Chen Hsieh, Dah-Yuu Lu

**Affiliations:** 1 Department of Pharmacology, School of Medicine, China Medical University, Taichung 404, Taiwan; u10201610@cmu.edu.tw (Y.-S.L.); u106216113@cmu.edu.tw (S.-W.L.); u108216088@cmu.edu.tw (H.-J.L.); 2 Department of Neurosurgery, Taichung Tzu Chi Hospital, Buddhist Tzu Chi Medical Foundation, Taichung 404, Taiwan; u100516123@cmu.edu.tw; 3 School of Medicine, Tzu Chi University, Taichung 404, Taiwan; 4 Department of Physiology, School of Medicine, China Medical University, Taichung 404, Taiwan; clin33@mail.cmu.edu.tw; 5 Graduate Institute of Biomedical Sciences, China Medical University, Taichung 404, Taiwan; u104116100@cmu.edu.tw; 6 Institute of New Drug Development, China Medical University, Taichung 404, Taiwan; u108216002@cmu.edu.tw; 7 Department of Pharmacy, China Medical University, Taichung 404, Taiwan; u108216203@cmu.edu.tw (C.-H.L.); ychsieh@cmu.edu.tw (Y.-C.H.); 8 Department of Photonics and Communication Engineering, Asia University, Taichung 404, Taiwan

**Keywords:** DRD2, glioblastoma, PD-L1, tumor-associated macrophage, paliperidone

## Abstract

**Simple Summary:**

The present study showed that a prescribed psychotropic medicine paliperidone inhibits GBM growth and prolongs survival in mouse brain tumor model and decreased the programmed death ligand 1 expression. Using the 3D co-culture also found that dopamine receptor D2 regulates the interaction of GBM-macrophage-induced PD-L1 expression in GBMs. In addition, the expression of DRD2 and PD-L1 in GBM modulates tumor-associated macrophage polarization. Our results also indicated that there is a contact-independent mechanism of PD-L1 induction in GBM upon interaction between GBM and monocytes. The present study also found that the interaction of GBM-macrophage-enhanced PD-L1 expression in GBM occurred by modulating the ERK and STAT3 signaling pathways. In addition, the inhibition of DRD2 reduces the upregulation of PD-1 expression, and it is regulating signaling in GBM.

**Abstract:**

A previous study from our group reported that monocyte adhesion to glioblastoma (GBM) promoted tumor growth and invasion activity and increased tumor-associated macrophages (TAMs) proliferation and inflammatory mediator secretion as well. The present study showed that prescribed psychotropic medicine paliperidone reduced GBM growth and immune checkpoint protein programmed death ligand (PD-L)1 expression and increased survival in an intracranial xenograft mouse model. An analysis of the database of patients with glioma showed that the levels of PD-L1 and dopamine receptor D (DRD)2 were higher in the GBM group than in the low grade astrocytoma and non-tumor groups. In addition, GFP expressing GBM (GBM-GFP) cells co-cultured with monocytes-differentiated macrophage enhanced PD-L1 expression in GBM cells. The enhancement of PD-L1 in GBM was antagonized by paliperidone and risperidone as well as DRD2 selective inhibitor L741426. The expression of CD206 (M2 phenotype marker) was observed to be markedly increased in bone marrow-derived macrophages (BMDMs) co-cultured with GBM. Importantly, treatment with paliperidone effectively decreased CD206 and also dramatically increased CD80 (M1 phenotype marker) in BMDMs. We have previously established a PD-L1 GBM-GFP cell line that stably expresses PD-L1. Experiments showed that the expressions of CD206 was increased and CD80 was mildly decreased in the BMDMs co-cultured with PD-L1 GBM-GFP cells. On the other hands, knockdown of DRD2 expression in GBM cells dramatically decreased the expression of CD206 but markedly increased CD80 expressions in BMDMs. The present study suggests that DRD2 may be involved in regulating the PD-L1 expression in GBM and the microenvironment of GBM. Our results provide a valuable therapeutic strategy and indicate that treatments combining DRD2 antagonist paliperidone with standard immunotherapy may be beneficial for GBM treatment.

## 1. Introduction

Glioblastoma (GBM) is considered the most deadly brain tumors because of its aggressive invasive growth and its resistance to chemotherapy and radiotherapy [1,2], and the average life expectancy of GBM patients is only approximately 15 months [3]. In histological analysis of GBM, a heterogeneous cellular composition of neoplastic glioma cells and non-neoplastic cells forms the tumor microenvironment that composes of brain-resident microglia, infiltrating monocytes/macrophages, reactive astrocytes, and other immune cell infiltrates [4]. Microglia and circulating monocytes/macrophages have been found to constitute up to 30% of tumor mass in both human and murine GBMs [5,6]. Accumulating studies indicated that non-neoplastic cells in the tumor microenvironment are important for the maintenance of cancer growth and the responses to therapies. Importantly, resident brain microglia and infiltrating blood monocytes are present in both low-grade and high-grade gliomas [7]. Monocytes belong to the mononuclear phagocyte system and arise from hematopoietic stem cells in the bone marrow that are released during infection and inflammation and differentiated into macrophages, which functions to maintain immunity homeostasis [8]. The most abundant populations of tumor-associated macrophages (TAM) in the glioma microenvironment include resident brain microglia, infiltrating blood monocytes, and circulating macrophages [7], which are the predominant inflammatory cells infiltrating the gliomas [9]. A previous study suggested that circulating monocytes that surround the tumor mass would differentiate into new cells and establish the tumor microenvironment [10]. Our previous study also reported that interaction of GBM and TAMs promoted GBM growth and invasion activity, as well as increased TAMs proliferation and inflammatory mediator secretion [11].

To date, many tumor entities show constant expression of programmed death (PD)-1, thereby evading immune surveillance [12,13]. Importantly, therapeutic strategies that aim at blocking immune checkpoint protein PD-1 or its ligand PD-L1 have proved successful [14]. PD-1 (also named CD279) and PD-L1 (also named CD274) have been recognized in mediating the immunosuppressive effects of tumors by promoting T effector cell dysfunction and the induction of regulatory T cells [15]. Clinically, similar effects have also been reported in other tumor entities, such as melanoma [16], lung [17], breast [18], and bladder [19]. PD-L1 has been observed to be overexpressed in GBM and GBM associated macrophages [20,21]. Recently, PD-L1 expression was observed in more than 88% of GBM cases, similar to other malignancies characterized by PD-L1 expression [22,23]. In addition, higher levels of PD-L1 expression are normally correlated with worse outcomes. In clinical studies, PD-L1 expression in patients with glioma was significantly higher in grade IV (GBM) than in grade II and III gliomas [24,25]. A previous study has reported that the levels of PD-L1 expression were positively correlated with the clinical grades of patients with gliomas [26]. Numerous studies have shown that PD-L1 expression in the glioma microenvironment contributes mainly by tumor-infiltrating myeloid cells such as macrophages, rather than the tumor cells themselves [27,28].

Epidemiologic studies have shown that patients with schizophrenia, who inherently have elevated dopamine receptor D (DRD)2 signaling, have an increased risk of cancers, while the risk returns to normal with DRD2 antagonists [29,30]. Antipsychotics and risperidone and its active metabolite, paliperidone, are DRD2 antagonists. They are FDA-approved clinical drugs for the treatment of schizophrenia [31]. Paliperidone has been recognized as the mildest psychiatric medicine for patients for tolerating clinical experience [32]. Comparison of tumor biopsies to matched normal brain tissues convincingly illustrated that DRD2 expression is significantly elevated in tumor tissues [33]. DRD2 has emerging as a novel therapeutic target in GBM and other cancers based on a series of studies that have demonstrated its selective overexpression in malignant tissues and the anticancer effects of its antagonism [34]. Importantly, GBM samples from patients have shown elevated synthesis and secretion of dopamine, which might initiate an autocrine signaling process within GBM microenvironment and, thus, contribute to tumor progression [35,36]. Activation of DRD2 has also been reported to induce GBM growth and shifts the cells to a stem cell-like state [37]. DRD2-targeting antipsychotics such as risperidone and haloperidol have been reported to induce apoptosis and reduce self-renewal in GBM cells [38]. Murine models of GBM exhibit a 14-fold increase in dopamine receptor expression compared to isogenic controls [39]. Furthermore, DRD2 silencing reduces U87 GBM growth by about 70–90% [33]. Therefore, inhibiting DRD2 signaling to treat GBM patients with tumors abundantly expressing DRD2 may be a promising therapeutic strategy.

The present study aims to investigate the molecular mechanisms underlying GBM and monocytes/macrophage interaction, as well as how DRD2 modulates PD-L1 induction within GBM and tumor microenvironment. The results further bring better understanding relative to the actual fate of GBM that recruits macrophages following infiltration and their roles in GBM progression. Our findings may help to identify therapeutic strategies that can be combined with standard glioma therapies.

## 2. Results

### 2.1. Paliperidone Inhibits GBM Growth in Mouse Brain Tumor Model

**Figure 1:**
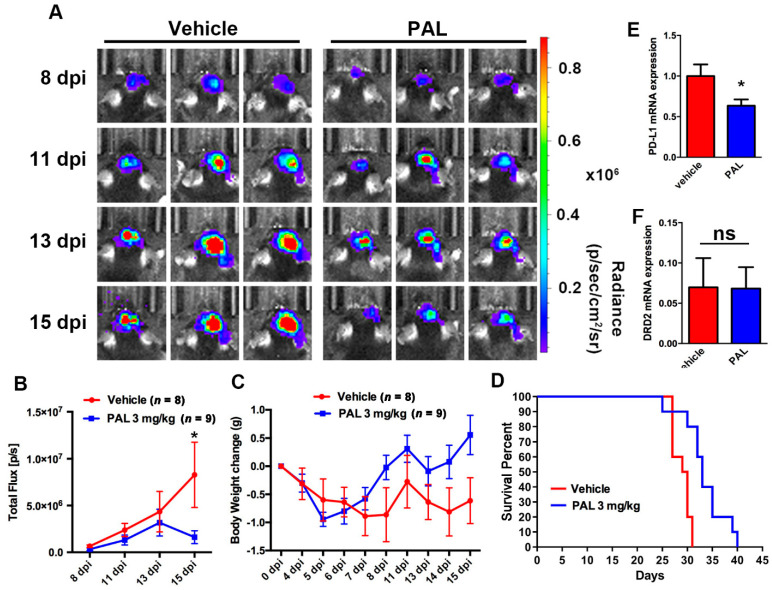
Paliperidone reduces GBM growth and PD-L1 expression and prolongs survival in the intracranial xenograft mouse model. ALTS1C1-luciferase GBM cells were implanted into mice brain, and the mouse was subjected to once daily intraperitoneal injection with paliperidone (PAL; 3 mg/kg) or vehicle. The tumor size was examined at the indicated time points (8, 11, 13, and 15 dpi) by IVIS image. Representative images from each group are shown (**A**). Quantification of tumor size is shown (**B**). The body weight changes are shown in (**C**). (**D**) Kaplan–Meier survival curve showed that mice with treated with paliperidone (PAL; 3 mg/kg) exhibited longer survival than the vehicle group (*p* = 0.02). Quantitative data are presented as mean ± SEMs.e.m. Messenger RNA levels of PD-L1 (**E**) and DRD2 (**F**) in the intracranial GBM were examined by real time PCR for day 24. * *p* < 0.05 compared with the vehicle control (Student’s t-test). Abbreviations: dpi: days post-implantation.

First, we used an intracranial xenograft model of ALTS1C1-Luc mouse GBM (stably expressing firefly luciferase), and the mouse was intraperitoneally administrated with paliperidone or vehicle once daily after 8 days post-implantation (d.p.i.). The IVIS images revealed an increase in tumor growth after 11 d.p.i. to 13 d.p.i. in the both vehicle and paliperidone groups. However, treatment with paliperidone markedly decreased the tumor volume than in the vehicle group (Figure 1A). Moreover, the quantification of tumor size by IVIS analysis showed that the tumor volumes were significantly increased in the vehicle group, while no increase between 13 and 15 dpi was observed in the paliperidone treatment group (Figure 1B). During these periods, the paliperidone treatment group showed a tendency in gaining weight (Figure 1C). In addition, the body weight loss of the vehicle group was observed. Consistent with imaging results, mice that were administrated with paliperidone possessed longer survival than the vehicle group (Figure 1D). Furthermore, analysis of the glioma in mouse brain showed that mRNA levels of PD-L1 were lower in the paliperidone treatment group than in the vehicle control group (Figure 1E). In addition, there was no significant difference of DRD2 between the paliperidone and the vehicle groups (Figure 1F). In addition, analysis of the GSE 4290 dataset of patients with glioma showed that levels of PD-L1 are higher in the GBM group than in the low-grade astrocytoma groups (grade II and grade III) and the non-tumor group (Figure S1A). The levels of DRD2 are also higher in the GBM group than in the grade II and III astrocytoma groups and the non-tumor group (Figure S1B).

### 2.2. Paliperidone Reduces PD-L1 Expression in GBM of GBM-Macrophage Co-Culture System

**Figure 2:**
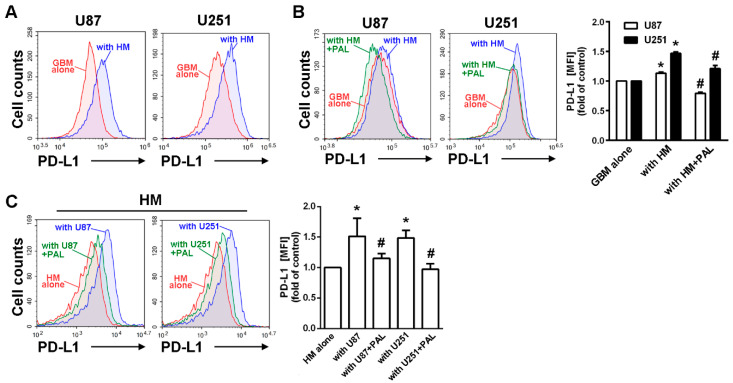
DRD2 involved in the PD-L1 expression in GBM cells in the GBM-macrophage co-culture system. (**A**) U87-GFP and U251-GFP cells were co-cultured with the THP-1 differentiated macrophage for 48 h. PD-L1 expression was determined by flow cytometry analysis. (**B**) U87-GFP and U251-GFP cells were treated with paliperidone (20 μM, PAL) for 30 min, and cells were then co-cultured with THP-1 macrophages (HM) after wash-out of paliperidone for 48 h. PD-L1 expression on GBM was determined by flow cytometry analysis. * *p* < 0.05 compared with GBM alone group. # *p* < 0.05 compared with THP-1 group. (**C**) THP-1 macrophages (HM) were treated with paliperidone (20 μM, PAL) for 30 min, washed out for 48 h, and then co-cultured with U87-GFP and U251-GFP cells. PD-L1 expression on cell surface of HM was determined by flow cytometry analysis. * *p* < 0.05 compared with THP-1 alone group. # *p* < 0.05 compared with U87 or U251 groups (*n* = 3–4).

According to the analysis of patients with glioma, we used an established human GBM-macrophage co-culture system to examine the effect of paliperidone in the interaction of monocytes and GBM [11]. GFP expressing GBM (GBM-GFP) cells were co-cultured with monocyte-differentiated macrophage, and then the expression of PD-L1 in GBM was determined. After 48 h co-culture, PD-L1 expression in the co-cultured GBM was higher than the GBM alone group (Figure 2A). In order to assess whether DRD2 was involved in the enhancement of PD-L1 expression, DRD2 clinical antagonist paliperidone (PAL) was administrated to the culture. As shown in Figure 2B, paliperidone decreased GBM-macrophage co-culture induced PD-L1 expression in GBM cells. In addition, paliperidone also decreased the levels of PD-L1 expression in monocyte-differentiated macrophages (Figure 2C). Moreover, treatment with PAL alone did not affect the PD-L1 expression in GBM (Figure S2A,B) or HM cells (Figure S2C).

### 2.3. Expression of DRD2 and PD-L1 in GBM Modulated Tumor-Associated Macrophage Polarization

**Figure 3:**
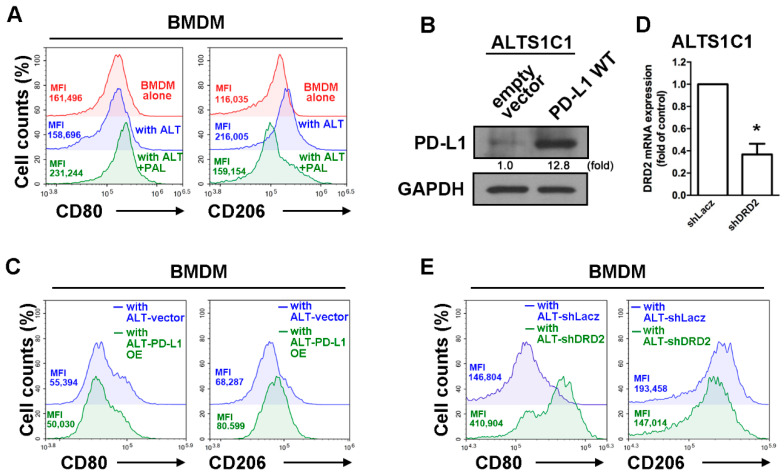
DRD2 and PD-L1 in GBM are involved in the polarization of TAMs in the GBM-macrophage co-cultured system. (**A**) BMDMs were co-cultured with ALTS1C1-GFP cells and then treated with or without PAL (20 μM) for 48 h. CD80 and CD206 expressions were determined by flow cytometry analysis. (**B**) Establishment of stable PD-L1 expression in ALTS1C1-GFP cells. The expression of PD-L1 was verified by Western blot. (**C**) BMDMs were co-cultured with PD-L1 ALTS1C1-GFP cells for 48 h. CD80 and CD206 were determined by flow cytometry analysis. (**D**) ALTS1C1-GFP cells were transfected with DRD2, and the expression of DRD2 was verified by real time-PCR. * *p* < 0.05 compared with shLacz group. (**E**) BMDMs were co-cultured with DRD2 knockdown of ALTS1C1-GFP cells for 48 h. CD80 and CD206 were determined by flow cytometry analysis.

Since the glioma in mouse brain showed that mRNA levels of PD-L1 were lower in the paliperidone treatment group, we further determined whether DRD2 expressed in mouse GBM changes the polarization of TAMs under interaction of GBM and macrophages. As shown in Figure 3A, the expression of CD206 was markedly increased in mouse bone marrow-derived macrophages (BMDMs) in the GBM-macrophage co-culture system. Importantly, treatment with paliperidone effectively decreased CD206 expression in BMDM. Furthermore, the expression of CD80 (M1 phenotype marker) on BMDMs was also dramatically increased by paliperidone treatment in GBM-macrophage co-cultured BMDM. In order to further determined whether DRD2 expression was involved in regulation of this phenomenon, we established a PD-L1 GBM-GFP cell line that stably expresses PD-L1 (Figure 3B). The expressions of CD206 increased while CD80 mildly decreased in the BMDMs co-cultured with PD-L1 GBM-GFP cells for 48 h (Figure 3C). On the other hands, GBM-GFP cells with transfected shRNA against DRD2 knock downed DRD2 expression (Figure 3D). The expressions of CD206 dramatically decreased, and CD80 markedly increased in the BMDMs co-cultured with GBM-GFP cells for 48 h (Figure 3E). In addition, treatment with PAL alone did not affect the expressions of both PD-L1 (Figure S2D) or the phenotype marker of macrophages (Figure S2E).

### 2.4. GBM-Primed Macrophage CM Effectively Induces PD-L1 Expression in GBM

**Figure 4:**
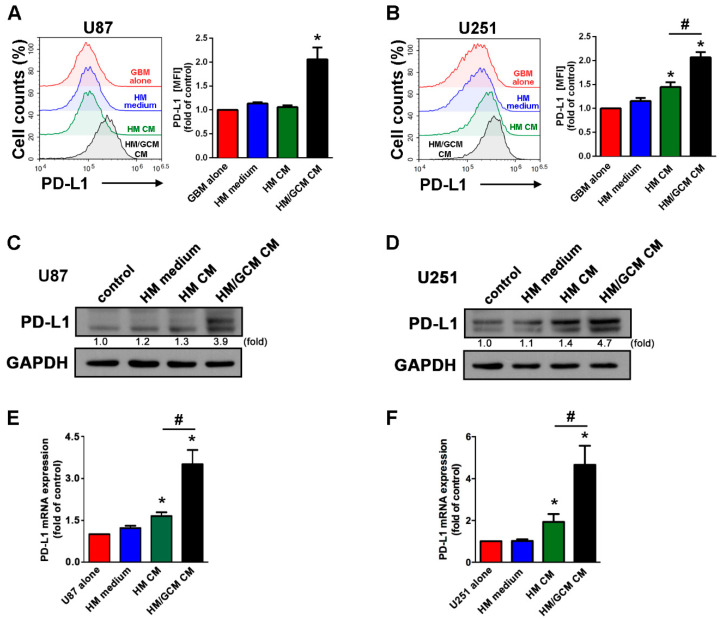
GBM primed macrophage potentiates PD-L1 expression in GBMs. Human U87 (**A**,**C**,**E**) and U251 (**B**,**D**,**F**) GBM were incubated with GBM cultured medium, THP-1 cultured medium, HM CM, or HM/GCM CM for 48 h. PD-L1 expression was determined by flow cytometry analysis (**A**,**B**), Western blot (**C** and **D**), and real time-PCR (**E**,**F**). * *p* < 0.05 compared with GBM alone group. # *p* < 0.05 compared with HM CM group. (*n* = 3–4).

In order to further examine whether upregulation of PD-L1 in GBM requires direct contact interaction with macrophages. We determined the expression of PD-L1 in GBM upon stimulation with conditioned medium (CM) from either differentiated human macrophages (HM CM) or GBM-CM (GCM)-primed human macrophages (HM/GCM CM). As shown in Figure 4A,B, HM/GCM CM significantly induced PD-L1 expression on both human U87 and U251 GBMs compared with treatment with HM medium or HM CM. In addition, the stimulation of HM CM only slightly increased PD-L1 expression in GBM (Figure 4B). In addition, the stimulation of HM/GCM CM also markedly induced PD-L1 protein expression in both human U87 (Figure 4C) and U251 GBMs (Figure 4D). The mRNA levels of PD-L1 in GBM also significantly increased after stimulation with HM/GCM CM than compared to HM CM (Figure 4E,F). In the meantime, there was only a mild increase in PD-L1 in GBM induced by HM CM. Our results suggest that the macrophage-induced PD-L1 expression on GBM does not require a direct contact type of interaction between GBM and macrophages. We also found that the CMs of macrophages after being primed by GCM had more potential than the CMs of naïve macrophages in inducing PD-L1 expression in GBM.

### 2.5. DRD2 Regulates the Interaction of GBM-Macrophage-Induced PD-L1 Expression in GBMs

**Figure 5:**
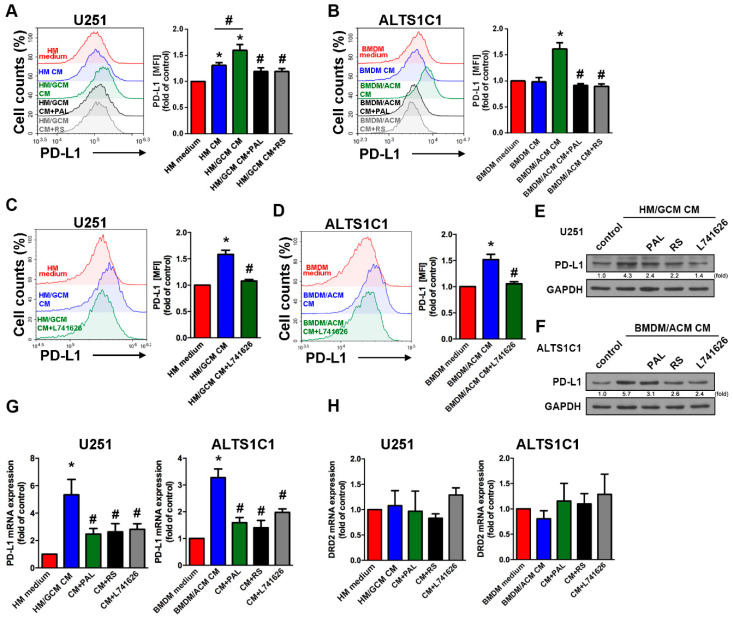
DRD2 is involved in the interaction of GBM-macrophage-induced PD-L1 expression in GBM. (**A**,**C**,**E**) Human U251 GBMs were incubated with human macrophage (HM) cultured medium, HM CM or HM/GCM CM, and then treated with paliperidone (20 μM, PAL), risperidone (20 μM, RS), or L741626 (1 μM) for 48 h. (**B**,**D**,**F**) Mouse ALTS1C1 GBMs were incubated with BMDMs cultured medium, BMDMs CM or BMDM/ACM CM, and then treated with or without PAL (20 μM), risperidone (20 μM, RS), or L741626 (1 μM) for 48 h. PD-L1 expression on the cell surface was determined by flow cytometry analysis (**A**–**D**) and Western blot (**E**,**F**). * *p* < 0.05 compared with HM medium group. # *p* < 0.05 compared with HM/GCM CM group. (*n* = 3–4) (**G**,**H**) mRNA levels of PD-L1 and DRD2 were determined by real-time PCR. * *p* < 0.05 compared with HM medium or BMDMs medium groups. # *p* < 0.05 compared with HM/GCM CM or BMDM/ACM CM groups (*n* = 3).

As shown in Figure 5A, treatment of clinical medicines such as paliperidone or risperidone decreased the enhancement of HM/GCM CM-induced PD-L1 expression in both human GBMs. Similarly, the administration of a DRD2 selective DRE2 antagonist L741626 also significantly decreased HM/GCM CM-induced PD-L1 expression in human GBM (Figure 5C). In addition, paliperidone, risperidone, or L741626 also effectively decreased BMDM/ACM CM-induced PD-L1 expression in mouse GBM (Figure 5B,D). Furthermore, the enhancements of PD-L1 protein expression in both human GBM (Figure 5E) and mouse GBM (Figure 5F) were reduced by paliperidone, risperidone, or L741626. Moreover, treatment with paliperidone, risperidone, or L741626 all markedly attenuated HM/GCM CM-enhanced or BMDM/ACM CM-enhanced mRNA of PD-L1 expression (Figure 5G) but did not affect mRNA of DRD2 expression (Figure 5H) in both human and mouse GBMs. In addition, the knockdown of DRD2 in GBM also reduced the enhancement of PD-L1 expression induced by HM/GCM (Figure S2F).

### 2.6. Involvement of ERK and STAT3 Signaling Pathways in the Interaction of GBM-Macrophage-Induced PD-L1 Expression in GBM

**Figure 6:**
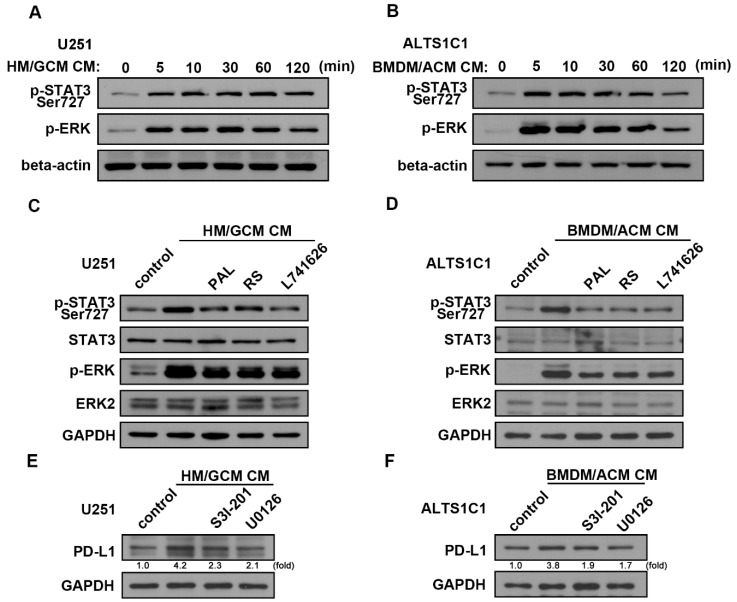
ERK and STAT3 signaling pathways are involved in the interaction of GBM-macrophage-induced PD-L1 expression in GBMs. Human U251 (**A**) and mouse ALTS1C1 (**B**) GBM were incubated with HM/GCM CM or BMDM/ACM CM for the indicated time periods (5, 10, 30, 60, or 120 min). Human U251 (**C**) and mouse ALTS1C1 (**D**) GBM were treated with PAL (20 μM), RS (20 μM), or L741626 (1 μM) and incubated with HM/GCM CM or BMDM/ACM CM. Phosphorylated-STAT3 and phosphorylated ERK expressions were determined by using Western blot analysis. Human U251 (**E**) and mouse ALTS1C1 (**F**) GBM were treated with S31-201 (30 μM) or U0126 (1 μM) and incubated with HM/GCM CM or BMDM/ACM CM for 48 h. PD-L1 expression was determined by using Western blot analysis. (*n* = 3–4).

We further determined the regulatory mechanism of PD-L1 expression under GBM-macrophage interaction and whether DRD2 modulates PD-L1 expression in GBM through these signaling pathways. As shown in Figure 6A, HM/GCM CM-induced increased phosphorylation of STAT3 and ERK in human GBM. Similarly, BMDM/ACM CM also increased STAT3 and ERK phosphorylation in mouse GBM (Figure 6B). Moreover, the enhancements of HM/GCM CM-induced or BMDM/ACM CM-induced phosphorylated STAT3 expression were reduced by PAL, RS, or L741626 treatment in human GBM (Figure 6C) and mouse GBM (Figure 6D). Furthermore, the enhancements of HM/GCM CM-induced or BMDM/ACM CM-induced PD-L1 expression in GBM were reduced by treatment with STAT3 inhibitor (S31-201) or MEK/ERK inhibitor (U0126) in human GBM (Figure 6E) and mouse GBM (Figure 6F). These results indicated that there is a contact-independent mechanism of PD-L1 induction in GBM upon interaction between GBM and monocytes. The interactions of GBM-macrophage-enhanced PD-L1 expression in GBM were by modulating the ERK and STAT3 signaling pathways. Our results also indicated that inhibition of DRD2 reduces the upregulation of PD-1 expression and its regulating signaling in GBM.

## 3. Discussion

Our previous study showed that in direct contact interactions of monocyte and GBM in a GBM-monocyte co-culture system, increased TAMs proliferation and elevated inflammatory factor secretion were observed [11], which resulted in gliomagenesis and tumor invasion [11]. As far as TAMs have been studied, the TAMs are classified as M1 or M2, which have also been identified to possess anti-tumoral or pro-tumoral activities based on the distinct characteristics of macrophages [40]. M2-TAMs are poor antigen presenters but can suppress T cell activation [41]. When more M2-(pro-tumor) macrophages were found in tumor sites, they increased in number as the tumor progresses [42]. Importantly, a clinical study has also reported that the TAMs in lower grade glioma were strongly manifested with M1 phenotype [43]. Importantly, a recent report also indicated that decreasing the tumor-promoting effects of macrophages in GBM could be considered as a potential strategy for a combination treatment with standard glioma therapies [44]. Recently, it has shown that ACT001, an ancient anti-inflammatory drug, reduces the expression of PD-L1 in glioblastoma, decreases M2 marker, and yet increases M1 marker expressions [45]. Many studies reported that there is a contact-free mechanism between tumor and tumor-associated cells which induces the immune check point up-regulation. In classical Hodgkin lymphoma, PD-L1 expression was elevated in monocytes co-cultured with Hodgkin and Reed–Sternberg (HRS) cells, but not in monocytes cultured with the supernatants of HRS cells [46]. PD-L1 expression increased on mouse melanoma cells by direct contact with CD11b positive bone marrow derived stromal cells [47]. Importantly, PD-L1 elevation in monocytes was observed after culturing with bladder cancer, which did not require direct contact but required soluble factors secreted by tumor cells [48]. The present study reported that there is a cell–cell contact-independent mechanism of PD-L1 expression in GBM underlying the interaction between GBM and TAMs, and DRD2 is involved in modulating the PD-L1 induction between GBM and its tumor microenvironment.

Many anticancer drugs that target PD-1, such as pembrolizumab (Keytruda^®^, Merck KGaA, Darmstadt, Germany) and nivolumab (Opdivo^®^, Bristol-Myers Squibb company, New York, NY, USA), have been approved by the Food and Drug Administration (FDA) in 2014. However, clinical trials of PD-1/PD-L1 antibody immunotherapy for glioma are relatively delayed and largely remained in phase two (pidilizumab) and phase three (nivolumab) [49,50]. In addition, two distinct studies, one involving 976 and the other 1052 glioma patients analyzing data from The Cancer Genome Atlas (TCGA) and Chinese Glioma Genome Atlas (CGGA), found that high PD-L1 mRNA expression levels were associated with significant shorter overall survival of glioma patients [24,51]. The increase in PD-L1+ TAMs surrounding glioma cells is associated with strong immune inhibition [27,52]. It has been reported that lower PD-L1 levels in glioma cells are associated with neither abating immune inhibition nor better prognosis of glioma, which is probably due to the linking with elevated PD-L1+ TAMs in the glioma microenvironment [53]. A recent study also indicated that increased dosage of PD-L1 antibody crossed the blood–brain barrier in order to reach both brain and glioma and, thus, could effectively suppress GBM growth by activating glioma-infiltrating T cells [54]. Our previous report found that monocytes that interacted with GBM could promote cell proliferation of TAMs and enhance cancer motility and gliomagenesis [11]. The present study further provides information about GBM–TAMs interaction that upregulates PD-L1 expression in GBM and also demonstrated that PD-L1 overexpressing in GBM enhances the polarization of TAMs toward the M2 phenotype.

In a clinical study, DRD2 mRNA and protein expressions significantly increased in GBM samples from human patient biopsies [55]. DRD2 and EGFR have been found to synergistically promote GBM tumors growth, and a combined inhibition of both DRD2 and EGFR causes a synergistic tumor-killing effect in mouse GBM [32]. Furthermore, combinations of temozolomide (TMZ) and DRD2 antagonists inhibit TMZ-induced protective autophagy and exert synergistic suppression of GBM growth [56]. Importantly, the immunomodulatory activity of the DRD2 antagonist ONC201 has recently been reported to involve increased circulation and activation of intratumoral natural killer cells, which may contribute to the antitumor effect exerted by this compound [57,58]. In fact, ONC201 is currently being assessed for the therapeutic treatment of recurrent glioblastoma in a phase two study [59]. Importantly, it appears from a recent clinical study that weekly oral administration of the DRD2 antagonist ONC201 is well tolerated in a subset of patients with recurrent GBM [60]. Our results support the previous findings that the inhibition of DRD2 by paliperidone reduces GBM growth and prolongs survival in the mouse brain and decreased PD-L1 expression in GBM as well. We also found that knockdown of DRD2 in GBM reverses the polarization of M2 phenotype toward M1 phenotype in TAMs.

STAT3 activates target genes that are thought to promote tumor progression, including cell proliferation, migration, and invasion [61]. Furthermore, STAT3 is found to be overexpressed in GBM. The activation of STAT3 is associated with poor prognosis in patients with glioma [62,63,64]. The administration of a STAT3 pharmacological inhibitor attenuated the infiltration of monocytes into glioma [65]. Importantly, a recent report also demonstrated that the ancient anti-inflammatory drug ACT001 reduces the expression of PD-L1 in glioblastoma by inhibiting the phosphorylation of STAT3 [45]. We previously reported that STAT3 signaling is involved in GBM-associated adhesion molecule expression and in monocyte adhesion to GBM [11]. Our previous report has also shown that bradykinin-induced IL-8 expressions and subsequent GBM migration are regulated by STAT3 signaling [66]. In addition, myeloid cell-associated PD-L1 induction in GBM was also reported to occur through a STAT3-dependent mechanism [67]. Similarly, activation of STAT3 and ERK pathways appeared to cause PD-L1 expression in lung adenocarcinoma [68]. Recently, ERK1/2 and STAT3 signaling pathways have also been reported to play critical roles in glioma invasiveness [69]. ERK signaling has also been shown to regulate PD-L1-associated GBM cell malignancy and aggressiveness [70]. It has also been reported that the activation of ERK signaling inhibits the degradation of PD-L1 and maintains stability of PD-L1 in GBM [71]. In a clinicopathological analysis, STAT3, ERK, and PD-L1 are associated with the progression of dermatofibrosarcoma protuberans [72]. The present study supports the previous studies that PD-L1 expression in GBM is modulated by STAT3 and ERK signaling pathways.

## 4. Materials and Methods

### 4.1. Materials

Paliperidone (DRD2 antagonist) was obtained from Toronto Research Chemicals (North York, ON, Canada). Risperidone (DRD2 antagonist), L741626 (DRD2 inhibitor), and S3I-201 (STAT3 inhibitor) were obtained from Sigma-Aldrich (St. Louis, MO, USA). U0126 (MEK1/2 inhibitor) was obtained from Calbiochem (San Diego, CA, USA). Primary antibodies for beta-actin (sc-47778), ERK2 (D-2), STAT3 (C-20), p-ERK (E-4), and p-STAT3 (sc-8001-RC768) were obtained from Santa Cruz Biotechnology (Santa Cruz, CA, USA). Primary antibodies for PD-L1 (13684) were obtained from Cell Signaling Technology (Danvers, MA, USA). Primary antibodies for GAPDH (SI-G8795) were obtained from Sigma-Aldrich. The short hairpin (sh)RNAs against DRD2 and Lacz were purchased from National RNAi Core Facility (NRC) (Academia Sinica, Taipei, Taiwan).

### 4.2. Animals

Male 8 week old C57BL/6 mice were obtained from the National Laboratory Animal Center (Taipei, Taiwan) and housed under standard laboratory conditions (21 ± 2 °C, 12 h L/D cycle, with food and water available ad libitum). All animal procedures were performed in accordance with the Institutional Animal Care and Use Committee (IACUC) of China Medical University (Taichung, Taiwan) (CMUIACUC-2019-139 and CMUIACUC-2021-159). The mice were intracranially implanted with mouse GBM, and the tumor volume was examined by using IVIS image. When the mice were observed with tumor growing out of the skull or with developed neurological symptoms, such as moribund state, these were indicators for sacrifice [73].

### 4.3. Intracranial Mouse Glioblastoma (GBM) Injection

C57BL/6 male mice (8 week of age) were obtained from the National Laboratory Animal Center (Taipei, Taiwan) and housed under standard laboratory conditions (21 ± 2 °C, 12 h light-dark cycle with food and water available ad libitum). Mice were anesthetized and placed in a stereotactic frame, and the skulls were exposed by incision. ALTS1C1 GBMs were freshly prepared and adjusted to 1 × 10^5^ cells/mL before implantation. In each skull, a hole was made 0.8 mm anterior and 2.5 mm to the right of the bregma, and 2 μl of GBM cells was injected using a 10 μL Hamilton syringe with a 26S-gage needle mounted in a stereotactic holder. The syringes were lowered at a depth of 3.5 mm, and the cells were injected at a rate of 0.4 μL/min. After intracranial implantation, a 5 min waiting period was observed before slowly withdrawing the syringe to prevent any reflux. The skull was then cleaned, and the incision was sutured. Tumors were allowed to grow, and the tumor volume was examined by using IVIS image once every four days for tumor progression. Luciferin (Caliper, Hopkinton, MA, USA) was intraperitoneally injected 5 mg per mouse. The mice were subjected to once daily intraperitoneal injection with paliperidone (PAL; 3 mg/kg) or vehicle until neurological symptoms became evident.

### 4.4. Cell Culture

Human glioma U251 cells were purchased from the (JCRB NO. IFO50288, Tokyo, Japan). Human glioma U87 cells, mouse glioma ALTS1C1 cells, and human monocyte THP-1 were purchased from the Bioresource Collection and Research Center (BCRC No. 60360, 60582 and 60430; Hsinchu, Taiwan). U251 and U87 cells were maintained with Minimum Essential Medium (MEM), ALTS1C1 cells were maintained with Dulbecco’s Modified Eagle Medium (DMEM), and THP-1 was maintained with RPMI-1640 medium. All the culture cells were grown in medium containing 10% fetal bovine serum (FBS), 100 mg/mL streptomycin, and 100 U/mL penicillin (PS). All the cells were incubated at 37 °C in a humidified atmosphere containing 5% CO_2_ and 95% air.

### 4.5. Differentiation of Human Monocyte and Mouse BMDMs

For human monocyte-differentiated macrophage, 2 × 10^6^ of THP-1 cells was differentiated with 100 nM phorbol 12-myristate 13-acetate (PMA) in 10 mL RPMI-1640 medium for 3 days. The differentiation of PMA treated cells was enhanced after initial 3 days stimulus by removing the PMA-containing medium and then incubating the cells in fresh RPMI-1640 for a further 1 days.

For bone marrow-derived macrophage (BMDM) differentiation, 2 × 10^6^ of bone marrow cells from the femur and tibiae of C57BL/6 was differentiated in 10 mL macrophage complete medium (20% L929 conditioned medium, 10% FBS in DMEM/F12 medium) for 6 days. On day 3, another 5 mL of macrophage complete medium was added to each dish for a further 3 days. For M2-type BMDMs differentiation, the mature BMDMs are then treated with 20 ng/mL IL-4 and 20 ng/mL IL-13 for 48 h.

### 4.6. GBM-Macrophage Co-Culture System

The co-culture experiments were performed according to our previous study [11]. U251-GFP and U87-GFP were seeded at 24-well plates (2 × 105 cells/well) in 150 μL phenol red free Matrigel (Corning, New York, NY, USA) and maintained in a culture medium condition for 24 h. Then, they were incubated with THP-1 cells (2 × 10^5^/well), and the wells were gently washed with serum-free medium removing non-adherent THP-1 cells. After 24 h, 3D co-cultured cells were extracted from Matrigel by using Cell Recovery Solution (Corning) according to the manufacturer’s instructions. The 3D co-cultured cells were isolated in single cell using 1 mg/mL Collagenase/Dispase (Roche, Basel, Switzerland) in culture medium and stirred slowly at 37 °C for 1 h in the dark. After cell separation, the cells were washed with serum-free medium, and flow cytometry analysis was undertaken.

### 4.7. Preparation of Conditioned Medium

Cells from human U251 GBM, mouse ALTS1C1 GBM, human macrophage, or BMDMs were cultivated in 10 mL complete medium into 10 cm dishes for 24 h, and then the culture medium was changed to serum free media. Conditioned medium (CM) was collected on days 1 and 2 and centrifuged at 1500 rpm for 10 min. After centrifugation, the supernatants were concentrated (10×, 10 kDa filter) and stored at −80 °C.

### 4.8. Cell Transfection

The protocol of cell transfection was performed according to our previous study [74]. GBM cells were cultured on a 6-well plate (1 × 105 cells/well) for 24 h and then replaced with serum-free MEM medium (1 mL/well) containing either plasmids (1 μg/mL) or shRNA (1 ng/mL) that had been pre-incubated with Lipofectamine 3000 (1 μL/mL) (LF3000; Invitrogen, (Carlsbad, CA, USA)) for 20 min. After transfection at 37 °C for 24 h, LF3000-containing medium was replaced with fresh serum-free MEM medium. The transfection efficiency was more than 60% [75].

### 4.9. Flow Cytometry Analysis

For co-culture THP-1 macrophage or BMDMs from brain tumor, the flow cytometric analysis is based on a BD Cytofix/Cytoperm Plus fixation/permeabilization protocol (BD Biosciences, San Jose, CA, USA). Cells were blocked for nonspecific binding by using anti-CD16/CD32 antibody (eBioscience, San Diego, CA, USA) for 20 min, and the cells were fixed and permeabilized with BD Cytofix/Cytoperm solution for 20 min at 4 °C. Cells were washed with BD Perm/Wash buffer, re-suspended in BD Perm/Wash buffer, and incubated with either anti-CD45-PerCP (BD Biosciences, 550994), anti-CD11b-FITC (11-0118, BioLegend, San Diego, CA, USA), anti-CD206-PE (h: eBioscience, 12-2069; m: Biolegend, 141705) anti-CD80-APC (m: eBioscience, 1957039), anti-PD-L1-APC (h: Biolegend, 329708; m: Biolegend, 124312), and isotype IgG control (eBioscience) for 30 min at 4 °C. The cells were then washed with Perm/Wash buffer and re-suspended in FACS buffer.

### 4.10. Western Blot Analysis

Cells were lysed on ice with radioimmunoprecipitation assay buffer for 30 min. Protein samples were separated by SDS-PAGE and then transferred to polyvinylidene fluoride (PVDF) membranes. The membranes were blocked with nonfat milk in PBS (5%) for 1 h and then probed with a primary antibody at 4 °C overnight. After undergoing several washes, the membranes were incubated with secondary antibodies. The blots were visualized by enhanced chemiluminescence with Kodak X-OMAT LS film (Eastman Kodak, Rochester, NY, USA). Quantitative results were obtained by using a computing densitometer and ImageQuant software (Molecular Dynamics, Sunnyvale, CA, USA). All the whole western blot figures can be found in the supplementary files.

### 4.11. RNA Extraction and Quantitative Real-Time PCR

The total RNA was extracted from tissues or cells using TRIzol reagent (Sigma-Aldrich, St. Louis, MO, USA) and then quantified using the BioDrop spectrophotometer (Cambridge, UK). The reverse transcription (RT) reaction was performed using the total RNA (2 μg) converted into cDNA by using the Invitrogen RT Kit (Carlsbad, CA, USA). Quantitative real-time PCR was performed with the StepOne Plus System (Applied Biosystems) using SYBR Green Master Mix (Applied Biosystems, Foster City, CA, USA). The PCR reactions were performed as follows: 45 cycles at 95 °C for 1 s and 60 °C for 20 s. The gene expression of beta-actin was used as an internal control. The threshold was set within the linear phase of target gene amplification in order to calculate the cycle number (denoted as CT).

### 4.12. Statistical Analysis

Statistical analysis was performed using GraphPad Prism 6 software (San Diego, CA, USA). Data are presented as mean ± SEM, and all experiments were performed in at least three independent replicates. Sample sizes are calculated to allow significance to be reached. Student’s *t*-test was used to determine statistical significance (*p* < 0.05). The survival of glioma-bearing mice was analyzed by Kaplan–Meier curves, and the differences were compared by log-rank test analysis.

## 5. Conclusions

Our results showed that inhibition of DRD2 by the clinical medicine paliperidone reduced GBM growth, prolonged survival, and decreased PD-L1 expression upon GBM-macrophage interaction. Moreover, PD-L1 overexpressing in GBM modulates TAMs’ polarization toward M2 phenotype, while the inhibition of DRD2 in GBM re-educates the polarization of TAMs toward the M1 phenotype. The present study also found that there is a cell–cell contact-independent mechanism of PD-L1 induction in GBM underlying the interaction of GBM and macrophage, and DRD2 also modulates the enhancement of PD-L1 expression in GBM. Our results may provide better understanding of the mechanism underlying how GBM interacts with macrophages following recruitment and their effects in GBM progression. Our findings could also help in identifying therapeutic strategies that can be combined with standard glioma therapies.

## Data Availability

Data are contained within the article or Supplementary Material.

## Supplementary Materials

The following are available online at https://www.mdpi.com/article/10.3390/cancers13174357/s1, Figure S1: Gene expression of PD-L1 (A) and DRD2 (B) in patients with glioma, Figure S2 Effects of paliperidone treatment in GBMs and macrophage. U87 (A), U251 (B), HM (C) or BMDMs (D) were treated with PAL, PD-L1 expression were determined by flow cytometer. HM cells were treated with PAL, CD206 expression was determined by flow cytometer (E). Human GBM cells was transfected with DRD2 shRNA and treated with HM/GCM, PD-L1 expression was determined by flow cytometer (F).
