# Supplementary material for: Paliperidone Inhibits Glioblastoma Growth in Mouse Brain Tumor Model and Reduces PD-L1 Expression
Source: Cancers (Basel). 2021 Aug 28;13(17):4357. doi: 10.3390/cancers13174357 (PMC8430966; doi:10.3390/cancers13174357)
Supplement: Supplementary file 1 [file cancers-13-04357-s001.zip › cancers-1351895-supplementary.pdf]

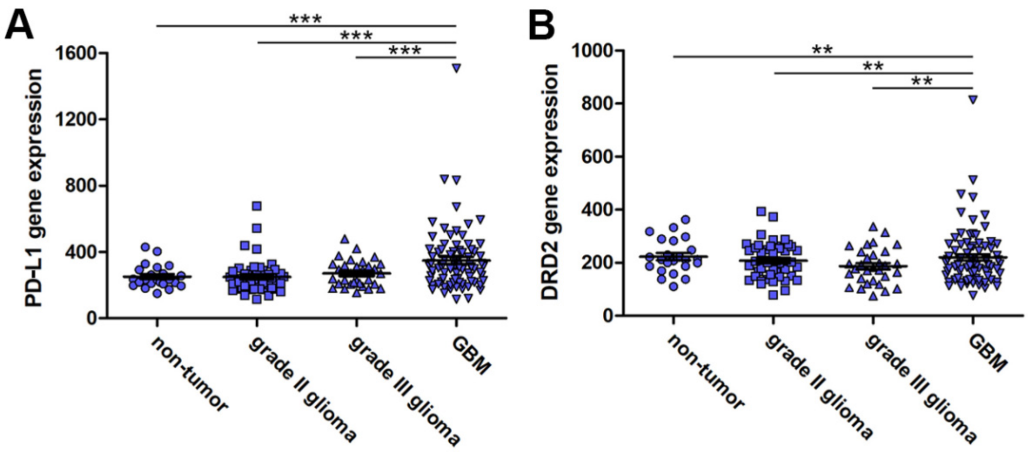

Figure S1 Gene expression of PD-L1 and DRD2 in patients with glioma.
Gene expression of PD-L1 (Fig. S1A) and DRD2 (Fig. S1B) in patients with glioma were analyzed by the GSE 4290 dataset.

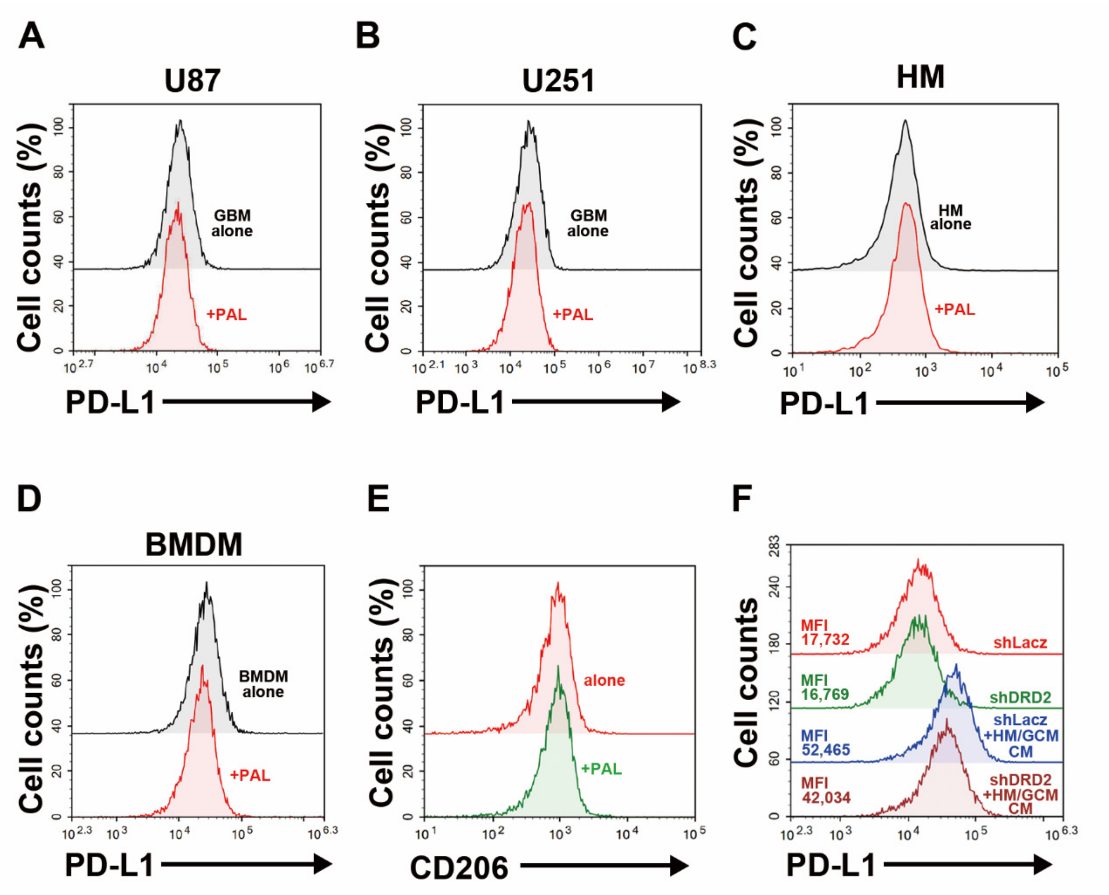

Figure S2 Effects of paliperidone treatment in GBMs and macrophage. U87 (A), U251 (B), HM (C) or BMDMs (D) were treated with PAL, PD-L1 expression were determined by flow cytometer. HM cells were treated with PAL, CD206 expression was determined by flow cytometer (E). Human GBM cells was transfected with DRD2 shRNA and treated with HM/GCM, PD-L1 expression was determined by flow cytometer (F).
