# Supplementary figures and images for: Paliperidone Inhibits Glioblastoma Growth in Mouse Brain Tumor Model and Reduces PD-L1 Expression
Source: Cancers (Basel). 2021 Aug 28;13(17):4357. doi: 10.3390/cancers13174357 (PMC8430966; doi:10.3390/cancers13174357)

Figure 4 CD

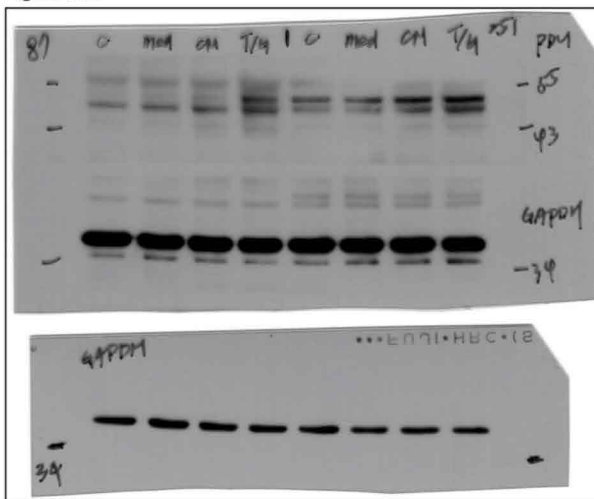

Figure 6A

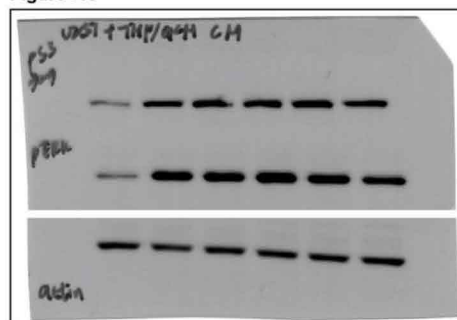

Figure 6B

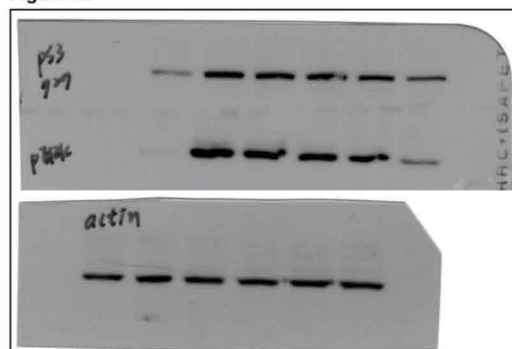

Figure 6C

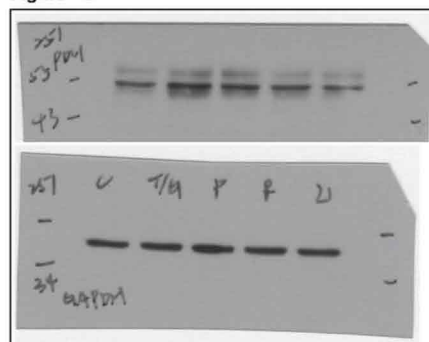

Figure 6D

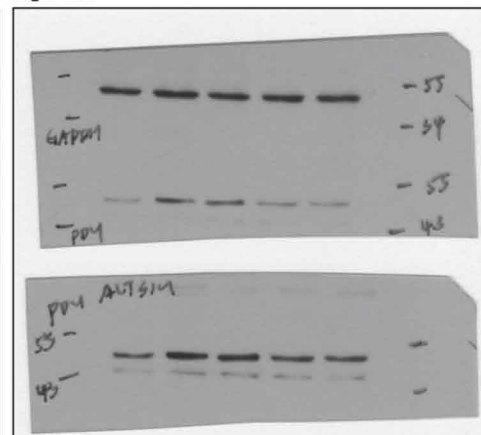

Figure 6E

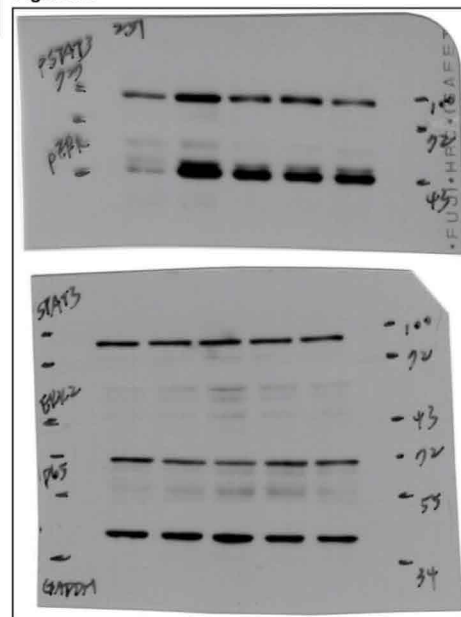

Figure 6F

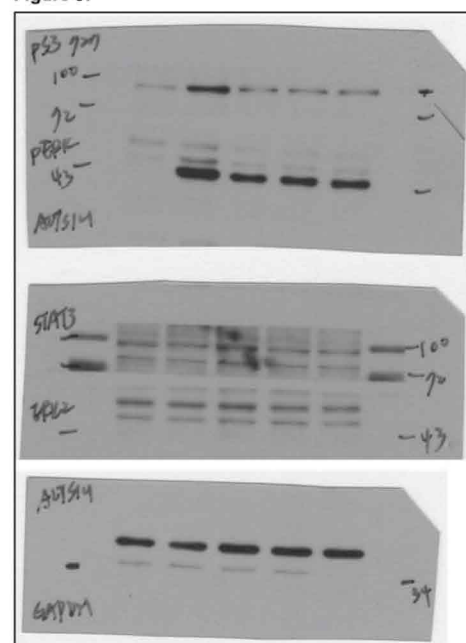

Figure 6G

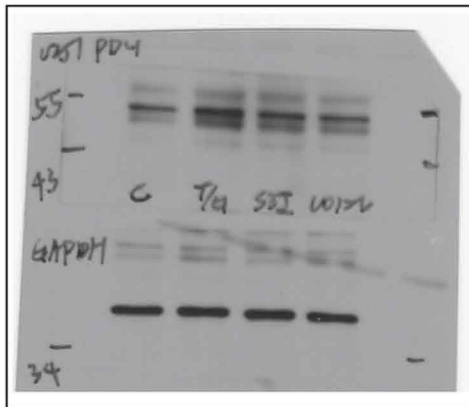

Figure 3B

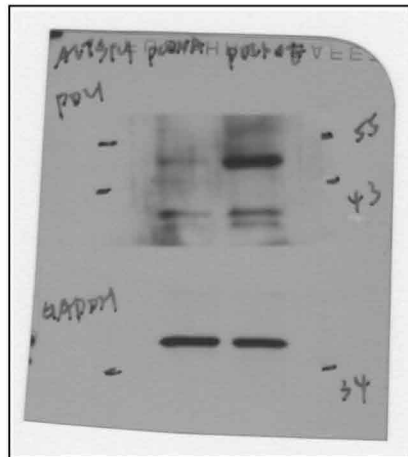

Figure 6H

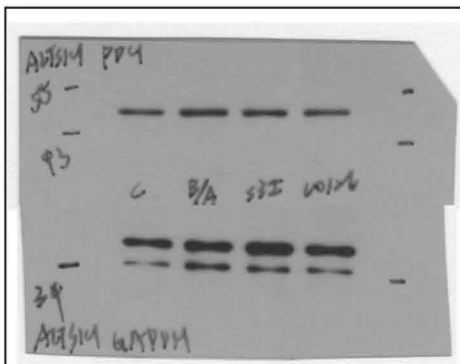

Supplement: Supplementary file 1 [file cancers-13-04357-s001.zip › cancers-1351895-original-images.pdf]
